# Climate Change, Emerging Vector-Borne Illnesses, and Anesthetic Considerations

**DOI:** 10.7759/cureus.57517

**Published:** 2024-04-03

**Authors:** Manuela Jaramillo Arias, Nikhil Kulkarni, Anh Le, Cheryl L Holder, Isik Unlu, Eugene S Fu

**Affiliations:** 1 Department of Anesthesiology, Herbert Wertheim College of Medicine, Miami, USA; 2 Department of Anesthesiology, University of Miami School of Medicine, Miami, USA; 3 Department of Internal Medicine, Herbert Wertheim College of Medicine, Miami, USA; 4 Internal Medicine, Jackson Memorial Hospital, Miami, USA; 5 Mosquito Control Division, Miami-Dade County, Miami, USA; 6 Anesthesiology, Jackson Memorial Hospital, Miami, USA

**Keywords:** vector-borne illness, anaphylaxis, malignant hyperthermia, vector borne diseases, anesthesia, climate change

## Abstract

As a result of the widespread prevalence of anesthetic usage, anesthesia-related complications are well studied, ranging from benign postoperative nausea and vomiting to potentially fatal complications, such as paralysis, malignant hyperthermia, and death. However, one intersection that still needs further analysis is the relationship between vector-borne illnesses (VBIs) and anesthetic complications. With the advent of climate change and global warming, what were previously endemic vectors have spread far beyond their typical regions, resulting in the spread of VBI. As the incidence of VBIs rapidly increases in the United States, operations for diagnostic testing, and thus the identification and treatments of these VBIs, have significantly diminished. A literature review was conducted to analyze case reports of patients with VBIs and anesthetic concerns with sources from PubMed and Google Scholar databases, and a wide range of complications were found.

## Introduction and background

Climate change is a pressing global concern, with far-reaching implications for human health, and Florida's unique subtropical climate and abundant water sources serve as a microcosm of the global struggle against the health implications of climate change. Diseases once considered distant threats now lurk in their backyard. This makes this state particularly vulnerable to the impacts of rising temperatures, shifting weather patterns, and the expansion of disease-carrying vectors. However, its impact on healthcare within the context of vector-borne illnesses (VBIs) and anesthesia remains an understudied area. Perioperative considerations in response to VBIs also remain inadequately explored. Healthcare professionals in Florida must recognize the growing threat posed by climate change and VBIs to adapt clinical practices to this new reality and to advocate for proactive measures that safeguard the health of patients and communities.

A literature review was conducted by identifying specific VBIs that pertain to endemic areas and isolating the following six VBIs, namely, malaria, Lyme disease, *Vibrio* species, dengue, Zika virus, and West Nile virus (WNV), and a relevant syndrome known as alpha-gal syndrome (AGS). Following the identification of our chosen VBI, multiple advanced searches were conducted in Google Scholar and PubMed using MeSH Terms (specifically highlighting "anesthesia" and the individual VBI), after which inclusion criteria were applied, such as free full text and publication date within 10 years. In the event that no pertinent publications were available with the prior criteria, the publication date limitation was removed, and all relevant articles were examined to assess their relevance to the article.

## Review

Climate change and VBIs

Expanding Vectors and VBIs in Florida

The first reported incident of isolated local mosquito-borne transmission and the first reported outbreak of Zika virus in the United States (US) occurred in Florida from June through August 2016 [1,2,3]. On June 26, 2023, the Florida Department of Health (FDOH) issued a statewide mosquito-borne illness advisory after the detection of local malaria in Sarasota County [4]. Since then, seven local cases of malaria were identified, subsequently treated, and have since recovered. Although the FDOH website does not currently report any presence of the Chikungunya virus in the state, a 2021 case report has already described a case of Chikungunya acquired in Florida with no international travel history [5]. According to the FDOH, dengue was eliminated from the US from 1934 until 2009 when a dengue outbreak was reported in Key West. In 2009-2010, an outbreak of dengue was identified in Key West [6]. WNV has been reported in all 67 of Florida's counties [7]. Many St. Louis encephalitis epidemics have been described in the US, with 223 reported cases in Florida in 1990, in a much colder climate [8].

Despite the rise in VBIs, in September 2021, the Centers for Disease Control and Prevention (CDC) suspended operations for diagnostic testing for most assays for parasitic diseases - over 20 diagnostic tests for parasitic infections. As of April 7, 2022, only three tests have resumed. The CDC has yet to provide dates for when they will restart the other tests. Beatty et al. recognized that the lapse in CDC laboratory services has created critical delays in the diagnosis and treatment of patients with suspected parasitic infections [9]. As a result, these research teams began to screen patients at high risk of Chagas infection. As of 2023, Beatty et al. screened approximately 700 people, with about 1% testing positive for Chagas, suggesting that about 18,000 to 20,000 people in the state live with this under-recognized disease [9]. Despite these findings, Chagas is not a reportable disease to the FDOH. Thus, the state is not monitoring its occurrence, and researchers are unable to track infections via routine blood and organ donor screening [10].

Climate change and disease-carrying vector expansion

Vectors, often arthropods, transmit infectious pathogens from infected hosts to uninfected individuals. Major global vector-borne diseases include malaria, dengue, chikungunya, yellow fever, Zika virus, and others, some of which have complex transmission involving both human and non-human hosts [11]. These VBIs are found from the tropics and subtropics to temperate climate zones. With rare exceptions, VBIs are not found in the colder regions of the world. The breadth of VBI transmission is influenced by two factors: the relative abundance of VBI-carrying vectors and the presence of the infecting parasite [12].

In a warmer world, ectothermic (cold-blooded) vectors thrive. The effects of changes in temperature, precipitation, humidity, and wind patterns have direct effects on these factors and are associated with changes in the reproduction, development rate, and longevity - and in turn the density- of these vectors. Furthermore, the development of a parasite accelerates as the temperature rises [11,12]. This further explains the invasion of such illnesses to locations that were not previously endemic [13].

How climate change can change disease burden

The effects of global climate change on infectious diseases will continue to be a major challenge until we have more scientific studies showing its effect. The emergence of most vector-borne diseases may be directly associated with the distribution and range expansion of vector populations. Rochlin et al. (2013) reported that with environmental conditions becoming more suitable for *Aedes albopictus*, this species population is expected to increase from 5% to 16% in the next two decades [1,14]. It is forecasted to increase to 43-49% by the end of the century. As the range of the vector species expands, vector-borne diseases are most likely to increase. *Aedes aegypti* and *Aedes albopictus* mosquitoes are invasive species with day-biting activity, which possess high disease vector potential. Their global distribution continues to expand and may move northward as global warming continues. These mosquitoes transmit the dengue virus, Zika, yellow fever, and chikungunya. When the temperature is higher to a certain point, faster metamorphosis and a shorter extrinsic incubation of these diseases may occur and may lead to epidemics in North America. Ecological studies will definitely help us to understand the possible consequences of global warming and its effect on vector-borne diseases [15].

Malaria has historically been one of the greatest public health problems on earth, infecting approximately 219 million people each year, resulting in an estimated 660,000 deaths [16]. Currently, there is growing concern that climate change and resistance to adulticides will change the distribution and burden of VBIs, potentially reversing the gains of control programs mainly focused on malaria control. Malaria transmission by *Anopheles gambiae* peaks at 25°C, while dengue transmission by *Aedes aegypti* peaks at 29°C. A 2020 paper hypothesized that that the direct effects of warming temperatures are likely to promote greater environmental suitability for dengue and other arbovirus transmission by *Aedes aegypti *and reduce suitability for malaria transmission by *Anopheles gambiae* [17]. We have already begun to see this change in burden - as of 2015, dengue has turned into the most rapidly expanding mosquito-borne infectious disease on earth [18]. The challenges faced in countries with a high burden of VBIs, such as foreshadowing the extent of the challenges that public health officials will face in Florida as the incidence of VBI continues to rise [19].

Seafood can be contaminated by *Vibrio* bacteria, another important cause of VBIs. Among these bacteria, *Vibrio cholerae*, *Vibrio parahaemolyticus*, and *Vibrio vulnificus* (Vv) are the primary species with pathogenic potential for humans [3,20]. *V. parahaemolyticus* and Vv are the leading causes of seafood-associated infections and mortality in the US [4,21]. Vv is spreading due to global warming [5,22]. Vv, typically attributed to vectors, such as raw shellfish and oysters, has begun to be associated with other vectors, such as undercooked fish and bee stings [6,23]. Liang et al. revealed that with the spread of Vv from seawater to freshwater environments, individuals could potentially contract the infection from insects, even when they have not directly interacted with contaminated water [7,23]. Traits from the zoonotic strains of Vv exhibit virulence in their primary hosts (iron-overloaded humans and healthy eels) and have recently been transmitted to the lineage causing human sepsis after the consumption of raw seafood [8,22]. Unsurprisingly, the CDC recently issued a warning about severe Vv infections in the US associated with warming coastal waters. Substantial and consistent evidence also supports the connections between *V. cholerae* and climatic factors [9,24].

Perioperative considerations for patients with VBIs

Malignant Hyperthermia VBIs

Under anesthesia care, side effects range from nausea and vomiting to potentially fatal side effects, like malignant hyperthermia (MH). MH is a complication of inhaled anesthetics or succinylcholine that occurs in patients who have a mutation in the ryanodine (RyR1) or dihydropyridine (DHPR) receptors; in these patients, the amount of calcium released from the sarcoplasmic reticulum is uncontrolled, resulting in sustained muscle contraction and the resulting complications (i.e., hyperthermia, rhabdomyolysis, and generalized muscle rigidity) [25]. A 2021 case report describes the case of a 29-year-old, previously healthy male in Sri Lanka with dengue and no other significant past medical history except a failed military fitness test because of a body temperature of 40°C and muscle cramp after running 1 km four years before. MH triggered by dengue fever had not previously been reported. In other reported cases and case series of awake MH, the triggers were sports activity, exercise, fever, and influenza A. As there were no other apparent triggering factors for MH, the patient was diagnosed as a case of awake or non-anesthetic MH triggered by dengue fever [26]. There is also growing evidence of a link between MH susceptibility and a history of exertional heat illness. This link suggests that certain individuals may carry a genetic vulnerability that makes them susceptible to hypermetabolic crisis, not only in the context of anesthesia or dengue but also in response to extreme heat and physical exertion [27]. This hypothesis is supported by current research on the impact of temperature on MH in animal studies [28-29]. These studies show that temperature plays a key role in MH sensitivity and that MH-sensitive mice were unable to survive for extended periods of time at 38°C compared to MH-resistant mice.

Anesthetic considerations for α-Gal syndrome

The red imported fire ant (RIFA) (*Solenopsis invicta*) is a non-native invasive species to America. Its population has rapidly spread northward in the US since it was imported from South America in the 1930s [30]. Although severe IgE-mediated anaphylactic reactions due to insect bites rarely occur, reactions to fire ant stings can range from uncomplicated stings to life-threatening anaphylactic reactions. Fire ant venom causes an immediate burning sensation, after which erythema and wheal formation occur within two hours in response to the local recruitment of eosinophils, neutrophils, and lymphocytes via an IgE-mediated response mechanism. In cases of anaphylactic reactions to fire ant bites, the cause is due to a hypersensitivity reaction in patients who have been sensitized via a previous ant sting [31]. Fire ants have been reported to be responsible for most of the allergic reactions secondary to ants in the US [32,33]. A 2021 study examining the effects of RIFA encounters in Taiwan (2.8%, 106 individuals) experienced anaphylactic shock [34]. A case report written in Texas described a 30-year-old woman who had a fatal anaphylactic reaction following multiple fire ant stings [35]. RIFA is not the only vector associated with anaphylaxis. The α-Gal syndrome, primarily linked to lone star tick bites in the US, is characterized by delayed allergic reactions to mammalian meat and elevated IgE levels to α-Gal. The primary treatment is dietary restriction. A minority of cases may benefit from avoiding a wide range of products containing mammalian-derived constituents, like gelatin [36]. Animal-derived components are frequently used in anesthesia and surgery without patient or provider awareness [37].

The α-Gal syndrome, primarily linked to lone star tick bites in the US, is characterized by delayed allergic reactions to mammalian meat and elevated IgE levels to α-Gal. The primary treatment is dietary restriction of mammalian meat and occasionally dairy products. Some patients may need to avoid mammalian-derived products, such as heart valves, gelatin-based plasma expanders, and pancreatic enzymes, but this pertains to specific patient groups. Some patients may benefit from avoiding a wide range of products containing mammalian-derived constituents, like gelatin [37]. Animal-derived components are frequently used in anesthesia and surgery without patient or provider awareness. Some medications contain non-therapeutic animal-derived substances, making up a substantial portion of the drug formulation. A study in the UK found that 74 out of the top 100 prescribed drugs contained animal-derived ingredients. Porcine-derived gelatin can be found in hydrocolloid dressings used in various surgeries. In addition, surgical materials like composite and biological mesh, biopolymer sutures, orthopedic spacers, heart valves, and hemostasis matrix may also contain animal-derived components, often with limited accessibility to this information. Special consideration should be taken when using these animal-derived components when treating patients with α-Gal syndrome [37].

Anesthetic considerations for malaria 

Malaria is among the most common parasitic illnesses in the world, affecting more than 240 million in 2021. Similar to the previously outlined viral vector illnesses discussed in this article, malaria is primarily distributed through the *Anopheles mosquito* genus, and the increasing global burden of the mosquito indicates that malaria has the potential to become even more prevalent in the future. Malaria affects multiple organ systems and thereby has many implications for management during general anesthesia. Factors to consider include the choice of anesthetic agents, intraoperative hemodynamic support, and possible relapse of malaria of specific species. Postoperative management of malaria patients depends on the severity of the patient, as additional resources ranging from ventilation support to multiorgan therapy may be indicated. However, it should be stated that there is not enough current literature outlining the usage of neuraxial anesthetic agents in patients with malaria [38]. For example, patients who suffer from cerebral malaria require maintenance of low intracranial pressure due to the blockage of cerebral vascular flow by parasitized erythrocytes [12,38]. In these patients, drugs such as ketamine, which increases cerebral blood flow and therefore ICP, must be avoided for drugs, such as propofol, that do not influence cerebral blood flow. It is also important to conduct extensive respiratory function assessment in patients with active malaria, as they are prone to the development of acute lung injury or adult respiratory distress syndrome. Acute renal failure has also been implicated in 30% of malaria cases, which can result in electrolyte abnormalities, such as hyponatremia or hyperkalemia, and fluid overload. Management of these patients includes electrolyte repletion, loop diuretics, and possibly preoperative dialysis. Another complication of malaria is hyperthermia in the intraoperative course, which can be mistaken for MH among patients without a prior diagnosis of malaria [39]. In malaria-endemic regions, recommendations to administer antimalarial agents to presurgical patients exist due to the immunosuppressive effects of anesthesia and surgery, which may result in active infection intra- and postoperatively among patients with quiescent parasitemia. Malaria has also been implicated in cases of intraoperative hyperpyrexia in malaria-endemic regions, calling into question the necessity for malaria screening in both symptomatic and asymptomatic patients [39].

Anesthetic considerations for Lyme

Lyme disease (LD), typically spread from *Ixodes* spp. or deer tick bites, is known to have a range of complications depending on the stage of infection. While early LD (stage 1) has primarily cutaneous and gastrointestinal issues, it is during the acute disseminated infection period (stage 2) where the infection could have anesthesia-related complications. There have been reports of Lyme carditis manifesting as high-degree atrioventricular blocks, suggesting the need for extensive cardiac workup in patients with a history of LD prior to operation. In addition, due to the transient nature of LD affecting the conduction system, patients should be closely monitored for ECG changes during the perioperative period. Another aspect to consider when managing patients with active LD is the choice of anesthetic agents, with volatile agents being reported to adversely affect the function of immune cells, thereby affecting the body's fight against infection [40].

Anesthetic considerations for *Vibrio*

Vv is one of the leading causes of seafood-associated infections and mortality in the US [13,21]. While most prevention efforts limiting the spread of Vv focus on oyster consumption and wound infection, Vv needs to be considered in the context of VBIs. One case describes that a 53-year-old male was reported to have contracted Vv after being stung by a bee [23]. He passed away only two days after admission. A few days later, the laboratory cultured Vv from his blood samples. Due to the quick progression of the disease course, 50-60% of *Vibrio* necrotizing fasciitis (VNF) cases present with septic shock and various organ dysfunctions on admission. In patients with VNF and septic shock, early initiation of simple incisions and drainage under regional anesthesia followed by complete debridement 24 hours later after patient optimization has been found to be more feasible and efficacious, when compared with the aggressive early surgical debridement strategy [41].

Pandemics, including cholera outbreaks in the past, have played a pivotal role in shaping the development of anesthesia and intensive care [14] medicine [15,42]. According to the CDC, 150-200 cases of the infections are reported each year, and about 20 people with the infection die [43]. Nevertheless, the global dissemination method of this disease remains uncertain. Evidence has correlated raw fish consumption or fish handling to a few cholera cases or cholera epidemics and warned that fish are reservoirs of *V. cholerae* [44]. During the intraoperative management of cholera patients, it is advisable to choose anesthesia agents with minimal respiratory depression, maintain spontaneous ventilation where feasible, and use neuromuscular blockers cautiously [45].

Anesthetic considerations for dengue

High heat and humidity associated with climate change increase mosquito activity as they are ideal conditions for breeding. Dengue fever is one of the mosquito-borne diseases that have plagued the developing world and has many anesthetic implications. Models have shown that climate change can increase the geographical area favorable for dengue transmission [46]. Dengue fever can present with symptoms ranging from mild to threatening without any effective treatment. Severe manifestations of dengue include altered vascular endothelial permeability, plasma leakage, thrombocytopenia, hypotension, and shock. Dengue hemorrhagic fever (DHF) and dengue shock syndrome are feared consequences of dengue illness. DHF can present diagnostic challenges in obstetric anesthesia due to the similar presentation to HELLP (hemolysis, elevated liver enzymes, and low platelets) syndrome, characterized by thrombocytopenia, albuminuria, and elevated liver enzymes.

One case report outlined the anesthetic management for a pregnant woman with dengue hemorrhagic fever, suggesting that general anesthesia may be safer than spinal anesthesia in these patients due to fluctuating platelet counts and the risk for spinal hematoma [47]. A 2017 trial found that in adult patients with dengue and thrombocytopenia, prophylactic platelet transfusion was not found to be superior to supportive care in preventing bleeding [48]. They also warned that it might be associated with adverse effects. An astute anesthesiologist should be aware of many ways active dengue infection can complicate the intraoperative course and derive a plan to support their patients. 

Anesthetic considerations for WNV

As previously mentioned, WNV is a VBI well-known for its potentially deadly side effect profile stemming from its neuroinvasive nature. In animal studies on the impact of halothane on WNV neuroinvasion and mortality in mice, it was found that halothane causes immunosuppression after 10 minutes exposure. More research must be conducted addressing the immunosuppressive nature of inhaled anesthetics and their potential implication in worsening viral infections. Another anesthetic consideration for WNV is the prevalence of acute flaccid paralysis of respiratory muscles leading to respiratory failure in 12% of patients with neuroinvasive WNV disease, which can complicate ventilation during general anesthesia [49].

Anesthetic considerations for Zika

The recent Zika virus outbreak among pregnant women in the US has generated a wider conversation on infection control during the peripartum period. Zika can be transmitted via sexual contact, mother to fetus, and blood. Patients infected with Zika virus have an increased risk for Guillain-Barre syndrome (GBS), which has many anesthetic considerations due to autonomic dysfunction, increased risk of thromboembolism, and aspiration [50]. The use of succinylcholine is contraindicated in patients with GBS, and a careful choice of anesthetic agents is required to prevent prolonged neuromuscular blockade. Newborns with Zika congenital syndrome can present with micrognathia and other vocal cord abnormalities, which can complicate general anesthesia via difficulty in bag-mask ventilation and obstructed viewing during direct laryngoscopy [51]. Anesthesiologists should be aware of complications of active Zika infection and plan accordingly. Most importantly, because of its blood-borne transmission nature, appropriate measures should be taken to prevent the spread of disease when performing neuraxial labor analgesia or anesthesia.

## Conclusions

The intersection of climate change and the expanding range of mosquito vectors in Florida poses significant challenges for anesthesia care and management in the Florida region. The heightened prevalence and activity of mosquito vectors, potentially carrying infectious diseases, such as dengue and Zika viruses, necessitate a reevaluation of anesthesia protocols and practices to mitigate the increased risk of VBIs. The rising temperatures and changing environmental conditions create an environment conducive to the proliferation of these vectors, demanding a proactive approach to adapting anesthesia strategies. Key challenges include the need for enhanced infection control measures, consideration of emerging diseases, and the development of protocols to address potential disruptions in healthcare delivery. Addressing these challenges is imperative for ensuring the safety and efficacy of anesthesia care in the face of evolving environmental conditions and vector dynamics in Florida.

Climate change is not solely an environmental concern but a multifaceted challenge to human health. As the earth’s temperature continues to rise, so does the prevalence of VBIs, resulting in potentially life-threatening complications, such as malignant hyperthermia. Case studies spanning the globe have documented the complexities associated with VBIs during the delivery of anesthesia, including instances of acute lung injuries occurring subsequent to platelet transfusions in patients with dengue fever. The emergence of the RIFA and the lone star tick, both having the potential to trigger severe anaphylactic reactions, further shed light on the practical challenges faced by healthcare providers in areas with a high burden of VBIs.

Florida's climate, its people, and America’s healthcare system are inextricably linked in this battle against climate-induced health risks. Our findings highlight the need for healthcare practitioners to adapt to the changing landscape of healthcare through interdisciplinary collaboration. They also emphasize the need for increased surveillance measures to better understand the prevalence and morbidity of VBIs in our community. Climate change considerations in perioperative management strategies are crucial to helping ensure the safety of anesthesia procedures and patient well-being.

## References

[1] Dobson GP (2020). Trauma of major surgery: a global problem that is not going away. Int J Surg.

[2] Likos A, Griffin I, Bingham AM (2016). Local mosquito-borne transmission of Zika virus - Miami-Dade and Broward Counties, Florida, June-August 2016. MMWR Morb Mortal Wkly Rep.

[3] Philip C, Novick CG, Novick LF (2019). Local transmission of Zika virus in Miami-Dade County: the Florida Department of Health rises to the challenge. J Public Health Manag Pract.

[4] (2023). The Florida Department of Health issues mosquito borne illnesses advisory | Florida Department of Health. https://www.floridahealth.gov/newsroom/2023/06/20230626-mosquito-borne-illnesses.pr.html.

[5] Ali AA, Bajric B, Isache CL, Maharaj RP (2021). Mosquito borne illness in a Floridian hiker. Am J Emerg Med.

[6] (2023). Dengue fever | Florida Department of Health. https://www.floridahealth.gov/diseases-and-conditions/dengue/.

[7] (2023). West Nile virus (WNV) | Florida Department of Health. https://www.floridahealth.gov/diseases-and-conditions/west-nile-virus/.

[8] St St (2023). St. Louis encephalitis (SLE) | Florida Department of Health. https://www.floridahealth.gov/diseases-and-conditions/st-louis-encephalitis/index.html.

[9] Beatty NL, Forsyth CJ, Gilman RH (2022). Neglected testing for neglected tropical diseases at the CDC. Am J Trop Med Hyg.

[10] (2023). Chagas disease | Florida Department of Health. https://www.floridahealth.gov/diseases-and-conditions/chagas/index.html#:~:text=Chagas%20disease%20is%20caused%20by,commonly%20known%20as%20kissing%20bugs)..

[11] Rocklöv J, Dubrow R (2020). Author correction: climate change: an enduring challenge for vector-borne disease prevention and control. Nat Immunol.

[12] Martens W, Jetten T, Rotmans J, Niessen L (1995). Climate change and vector-borne diseases: a global modelling perspective. Global Environ Change.

[13] Iwamura T, Guzman-Holst A, Murray KA (2020). Accelerating invasion potential of disease vector Aedes aegypti under climate change. Nat Commun.

[14] Rochlin I, Ninivaggi DV, Hutchinson ML, Farajollahi A (2013). Climate change and range expansion of the Asian tiger mosquito (Aedes albopictus) in Northeastern USA: implications for public health practitioners. PLoS One.

[15] Strickman D (2020). Chapter 10 - invasive mosquito species and potential introductions. Mosquitoes, Communities, and Public Health in Texas.

[16] (2023). Malaria | Florida Department of Health. https://www.floridahealth.gov/diseases-and-conditions/malaria/index.html.

[17] Mordecai EA, Ryan SJ, Caldwell JM, Shah MM, LaBeaud AD (2020). Climate change could shift disease burden from malaria to arboviruses in Africa. Lancet Planet Health.

[18] Schwartz LM, Halloran ME, Durbin AP, Longini IM Jr (2015). The dengue vaccine pipeline: implications for the future of dengue control. Vaccine.

[19] Fish D, Tesh RB, Guzman H (2021). Emergence potential of mosquito-borne arboviruses from the Florida Everglades. PLoS One.

[20] Bonnin-Jusserand M, Copin S, Le Bris C (2019). Vibrio species involved in seafood-borne outbreaks (Vibrio cholerae, V. parahaemolyticus and V. vulnificus): Review of microbiological versus recent molecular detection methods in seafood products. Crit Rev Food Sci Nutr.

[21] Elmahdi S, DaSilva LV, Parveen S (2016). Antibiotic resistance of Vibrio parahaemolyticus and Vibrio vulnificus in various countries: A review. Food Microbiol.

[22] Hernández-Cabanyero C, Lee CT, Tolosa-Enguis V (2019). Adaptation to host in Vibrio vulnificus, a zoonotic pathogen that causes septicemia in fish and humans. Environ Microbiol.

[23] Liang JH, Liang WH, Deng YQ, Fu ZG, Deng JL, Chen YH (2021). Vibrio vulnificus infection attributed to bee sting: a case report. Emerg Microbes Infect.

[24] Asadgol Z, Badirzadeh A, Niazi S, Mokhayeri Y, Kermani M, Mohammadi H, Gholami M (2020). How climate change can affect cholera incidence and prevalence? A systematic review. Environ Sci Pollut Res Int.

[25] Rosenberg H, Davis M, James D, Pollock N, Stowell K (2007). Malignant hyperthermia. Orphanet J Rare Dis.

[26] Madhusankha KH, Fernando H, Kumarasiri S, Liyanarachchi GG (2021). Dengue fever-triggered malignant hyperthermia. Cureus.

[27] Hopkins PM (2007). Is there a link between malignant hyperthermia and exertional heat illness?. Br J Sports Med.

[28] Lopez JR, Kaura V, Diggle CP, Hopkins PM, Allen PD (2018). Malignant hyperthermia, environmental heat stress, and intracellular calcium dysregulation in a mouse model expressing the p.G2435R variant of RYR1. Br J Anaesth.

[29] Kovats RS, Hajat S (2008). Heat stress and public health: a critical review. Annu Rev Public Health.

[30] Lytle AJ, Costa JT, Warren RJ 2nd (2020). Invasion and high-elevation acclimation of the red imported fire ant, Solenopsis invicta, in the southern Blue Ridge Escarpment region of North America. PLoS One.

[31] Singh S, Mann BK (2013). Insect bite reactions. Indian J Dermatol Venereol Leprol.

[32] Alsharani M, Alanazi M, Alsalamah M (2009). Black ant stings caused by Pachycondyla sennaarensis: a significant health hazard. Ann Saudi Med.

[33] Hoffman DR (1995). Fire ant venom allergy. Allergy.

[34] Liu YS, Huang SA, Lin IL, Lin CC, Lai HK, Yang CH, Huang RN (2021). Establishment and social impacts of the red imported fire ant, Solenopsis invicta, (Hymenoptera: Formicidae) in Taiwan. Int J Environ Res Public Health.

[35] Prahlow JA, Barnard JJ (1998). Fatal anaphylaxis due to fire ant stings. Am J Forensic Med Pathol.

[36] Platts-Mills TA, Li RC, Keshavarz B, Smith AR, Wilson JM (2020). Diagnosis and management of patients with the α-Gal syndrome. J Allergy Clin Immunol Pract.

[37] Rodger D, Blackshaw BP (2019). Using animal-derived constituents in anaesthesia and surgery: the case for disclosing to patients. BMC Med Ethics.

[38] Soltanifar D, Carvalho B, Sultan P (2015). Perioperative considerations of the patient with malaria. Can J Anaesth.

[39] Amanor-Boadu SD, Mohammed A (2003). The diagnostic dilemma of intraoperative hyperpyrexia in a malaria endemic area. West Afr J Med.

[40] Wan D, Blakely C, Branscombe P, Suarez-Fuster L, Glover B, Baranchuk A (2018). Lyme carditis and high-degree atrioventricular block. Am J Cardiol.

[41] Hong GL, Dai XQ, Lu CJ (2014). Temporizing surgical management improves outcome in patients with Vibrio necrotizing fasciitis complicated with septic shock on admission. Burns.

[42] Levin AB, Ball CM, Featherstone PJ (2020). From cholera to COVID-19: how pandemics have shaped the development of anaesthesia and intensive care medicine. Anaesth Intensive Care.

[43] Health Alert Network (HAN) - 00497 | Severe Vibrio vulnificus Infections in the United States Associated with Warming Coastal Waters. Accessed: September 21, 2023. https://emergency.cdc.gov/han/2023/han00497.asp..

[44] Halpern M, Izhaki I (2017). Fish as hosts of Vibrio cholerae. Front Microbiol.

[45] Ginosar Y, Shapira SC (1995). The role of an anaesthetist in a field hospital during the cholera epidemic among Rwandan refugees in Goma. Br J Anaesth.

[46] Hales S, De Wet N, Maindonald J, Woodward A (2002). Potential effect of population and climate changes on global distribution of dengue fever: an empirical model. Lancet.

[47] Ahmed U, Aman A (2022). Intraoperative post partum hemorrhage in a patient with dengue fever. Pak J Med Sci.

[48] Lye DC, Archuleta S, Syed-Omar SF (2017). Prophylactic platelet transfusion plus supportive care versus supportive care alone in adults with dengue and thrombocytopenia: a multicentre, open-label, randomised, superiority trial. Lancet.

[49] Sejvar JJ, Bode AV, Marfin AA (2005). West Nile virus-associated flaccid paralysis. Emerg Infect Dis.

[50] Parra B, Lizarazo J, Jiménez-Arango JA (2016). Guillain-Barré syndrome associated with Zika virus infection in Colombia. N Engl J Med.

[51] Tutiven JL, Pruden BT, Banks JS, Stevenson M, Birnbach DJ (2017). Zika virus: obstetric and pediatric anesthesia considerations. Anesth Analg.

